# Takeaway meal consumption and risk markers for coronary heart disease, type 2 diabetes and obesity in children aged 9–10 years: a cross-sectional study

**DOI:** 10.1136/archdischild-2017-312981

**Published:** 2017-12-03

**Authors:** Angela S Donin, Claire M Nightingale, Chris G Owen, Alicja R Rudnicka, Derek G Cook, Peter H Whincup

**Affiliations:** Population Health Research Institute, St George’s, University of London, London, UK

**Keywords:** nutrition, takeaway meals, cholesterol, coronary heart disease, diet

## Abstract

**Objective:**

To investigate associations between takeaway meal consumption and risk markers for coronary heart disease, type 2 diabetes and obesity risk markers in children.

**Design:**

A cross-sectional, school-based observational study.

**Setting:**

85 primary schools across London, Birmingham and Leicester.

**Participants:**

1948 UK primary school children in year 5, aged 9–10 years.

**Main outcome measures:**

Children reported their frequency of takeaway meal consumption, completed a 24-hour dietary recall, had physical measurements and provided a fasting blood sample.

**Results:**

Among 1948 participants with complete data, 499 (26%) never/hardly ever consumed a takeaway meal, 894 (46%) did so <1/week and 555 (28%) did ≥1/week. In models adjusted for age, sex, month, school, ethnicity and socioeconomic status, more frequent takeaway meal consumption was associated with higher dietary intakes of energy, fat % energy and saturated fat % energy and higher energy density (all P trend <0.001) and lower starch, protein and micronutrient intakes (all P trend <0.05). A higher frequency of takeaway meal consumption was associated with higher serum total cholesterol and low-density lipoprotein (LDL) cholesterol (P trend=0.04, 0.01, respectively); children eating a takeaway meal ≥1/week had total cholesterol and LDL cholesterol 0.09 mmol/L (95% CI 0.01 to 0.18) and 0.10 mmol/L (95% CI 0.02 to 0.18) higher respectively than children never/hardly ever eating a takeaway meal; their fat mass index was also higher.

**Conclusions:**

More frequent takeaway meal consumption in children was associated with unhealthy dietary nutrient intake patterns and potentially with adverse longer term consequences for obesity and coronary heart disease risk.

**What is already known?**

- Observational evidence suggests that adults who regularly eat takeaway meals have poorer diet quality, increased adiposity, type 2 diabetes and coronary heart disease.
- In the UK, takeaway meal consumption is increasing. However, little is known about the associations between takeaway meal consumption and chronic disease risk markers in children.

**What this study adds?**

- Our study suggests that children who regularly eat takeaway meals have adverse lipid profiles, higher body fat and poorer diet quality.
- Efforts to reduce takeaway meal consumption in children could have both short-term and long-term health benefits.

## Introduction

Previous studies in adults have reported that increased consumption of takeaway food is associated with increased body fatness,^1–3^ insulin resistance,^4^ type 2 diabetes^5^ and coronary heart disease (CHD)^6^; an association between takeaway meal consumption and obesity has also been reported in adolescents.^7^ Both adults and children who regularly eat takeaway food have poorer diet quality, higher total fat intakes and lower intakes of fruit and vegetables.^8–10^

In the UK, consumption of takeaway meals has increased. Between 1996 and 2006, the frequency of consumption has increased by more than a quarter, despite concerns about the high fat, sugar and salt content of many of these foods.^11^ A recent study in the UK reported that more than half of 11–14 year olds reported purchasing food from fast food outlets twice or more a week.^12^ The increasing consumption of takeaway meals is a particular concern in the context of high rates of childhood obesity^13^ and the early emergence of type 2 diabetes risks in children.^14^ However, the associations between frequency of takeaway meal consumption and risk markers for coronary heart disease and type 2 diabetes have been little studied in children.

We have therefore investigated the associations between frequency of takeaway meal consumption and risk markers for coronary heart disease, type 2 diabetes and obesity in a large multiethnic population of children. Using data from 24-hour dietary recalls, we have also examined the associations between reported frequency of takeaway meal consumption and nutrient intakes over a 24-hour period, and the nutrient content of the previous evening meal in relation to its source (prepared at home or obtained from a takeaway restaurant).

## Methods

### Participants

This investigation was based on the Child Heart And health Study in England (CHASE),^15^ which examined markers of cardiovascular disease and type 2 diabetes risk and their determinants in a multiethnic population of children aged 9–10 years. Parents or guardians provided informed written consent. Balanced numbers of children of South Asian, black African-Caribbean and white Europeans origin were invited to take part from a stratified random sample of 200 primary schools in London, Birmingham and Leicester. This investigation is based on the last 85 schools (visited between February 2006 and February 2007), in which detailed information on eating patterns and dietary nutrient intakes were collected (described in detail elsewhere^16^).

### Dietary assessment

Participants were asked about their eating patterns, including a question ‘how often do you have a meal from a takeaway restaurant?’ with four response options—never or hardly ever,<1/week, 1/week and >1/week. The interviewer made it clear that this only included meals from takeaway outlets and not convenience stores or supermarkets and included foods such as ‘burgers, fish and chips, Chinese, pizza and so on’ and not just drinks or snacks such as ‘crisps or fizzy drinks’. Because only a small number of children reported consuming a takeaway meal >1/week (n=129, 7% of the sample), the top two categories were combined for analysis. Dietary intake was assessed using a single, structured 24-hour recall including elements of the United States Department of Agriculture multiple pass method.^17^ Memory cues were used to aid recall, such as orientating the child on details of the previous day, and checking for any forgotten snacks or drinks that the child may have had through the day. Photographs of common foods were used to help the child estimate portion sizes. The children were also asked to report the source of each meal (eg, home, school, takeaway or ‘eat in’ restaurant). Energy and nutrient intakes were calculated by the Medical Research Council Human Nutrition Research centre (MRC-HNR) using an in-house food composition database.^18^ Energy density was calculated by dividing the reported total energy intake from food (kJ) by the total weight of food reported (g).^19^

### Physical measurements and blood sampling

Participating children had measurements of height (using a portable stadiometer, CMS Instruments, London, UK), weight (Tanita, Tokyo, Japan), waist circumference, multiple skinfold thicknesses and bioelectrical impedance, measured with a Bodystat 1500 body composition analyser (Bodystat Ltd, Isle of Man, UK). Bioelectrical impedance was used as the principal marker of body fat as it provides valid assessments in this ethnically diverse population.^20^ Fat-free mass was derived using validated equations, and fat mass index was calculated (kg/m^5^), which is independent of height.^21^ Seated blood pressure was measured twice in the right arm after a 5 min rest using an Omron 907 blood pressure recorder, with an appropriately sized cuff. Children provided fasting blood samples after an overnight fast for the measurement of all blood markers including total cholesterol and high-density lipoprotein (HDL) cholesterol and triglycerides; low-density lipoprotein (LDL) cholesterol was obtained using the Fredrickson-Friedewald equation.^22^ Serum insulin was measured using an ELISA method,^23^ plasma glucose was measured using the glucose oxidase method and  haemoglobin A1c was measured in whole blood by ion exchange high-performance liquid chromatography. The homeostasis model equations were used to provide an estimate of insulin resistance.^24^

### Ethnicity and socioeconomic status

Parents provided questionnaire-based information on their occupation(s), self-defined ethnicity and the ethnicity of the participating child. Participating children provided information on parental occupation(s) and on the place of birth of parents and grandparents. Parental occupation was coded using the National Statistics-Socioeconomic Classification (NS-SEC). If both parents worked, the highest occupational group recorded provided the basis of classification. NS-SEC was categorised into the three class version (professional and managerial, intermediate occupations, and routine and manual) plus categories of ‘economically inactive’ (currently unemployed or looking after the home) and ‘not classified’ (where the occupation was not stated). Ethnicity was defined using parental self-defined ethnicity for both parents, or using parentally defined child ethnicity. In a small number of participants where this information was not available (1.4%), child-defined place of origin of parents and grandparents was used to define ethnicity. The ‘other’ ethnic group includes all other categories of individual and mixed ethnic origins.

### Statistical methods

Statistical analyses were carried out using STATA/SE software (STATA/SE 12 for Windows, StataCorp LP, College Station, Texas, USA). Multilevel linear regression models were used to provide adjusted means by takeaway frequency groups for risk markers and dietary intake, using XTMIXED and LINCOM commands. All analyses were adjusted for sex, age in quartiles, ethnic group, socioeconomic status and month as fixed effects; school was fitted as a random effect to allow for the clustering of children within schools. In further analyses in which the influence of dietary saturated fat intake on differences in blood lipids by takeaway meal frequency was examined, classical measurement models (CME command) were used to allow for possible measurement error in dietary intake based on reassessment of dietary nutrient intakes in 86 study participants within a 1-year period of their original assessment.

## Results

Among 3679 children invited, 2529 (69% response rate) took part in the present study; participation rates were similar in all ethnic groups except the black African Caribbeans (66%). Among these 2529 children, analyses are based on 1948 children who provided full questionnaire data and had complete fasting blood samples; their mean age was 10.0 years (95% reference range 9.3 to 10.6 years), 1023 (52%) were girls. They included similar numbers of children of white European, black African-Caribbean, South Asian and other ethnic origins (n=475, 496, 495 and 482, respectively). The distribution of parental socioeconomic position included 26% in managerial/professional, 25% in intermediate and 29% in routine/manual occupations, with 16% economically inactive and 4% unclassified. The characteristics of participants eligible for inclusion in these analyses did not differ appreciably from those not included.

The frequencies of reported takeaway meal consumption are presented in the online supplementary table 1 by sex, ethnic group and socioeconomic status. Overall, 499 children (26%) reported never or hardly ever eating a takeaway meal, while 894 (46%) reported consuming a takeaway meal <1/week and 555 (28%) reported doing so ≥1/week. Boys reported consuming takeaway meals more often than girls (P=0.001) and children from lower socioeconomic groups also consumed takeaway meals more frequently than those from higher socioeconomic groups (P=0.008); there were no marked ethnic differences in frequency of takeaway meal consumption.

10.1136/archdischild-2017-312981.supp1 Supplementary Table 1

Table 1 presents the means and 95% CIs of risk markers for CHD and type 2 diabetes disease by frequency of takeaway meal consumption, adjusted for age (quartiles), sex, month, ethnic group, socioeconomic status and school (random effect), with formal tests for trend across the takeaway frequency groups. Fat mass index, sum of skinfolds, total cholesterol and LDL cholesterol all tended to be higher in children with a higher frequency of takeaway meals (P trend=0.03, 0.05, 0.04 and 0.01, respectively). Among children who reported consuming a takeaway meal ≥1/week, total cholesterol was 0.09 mmol/L (95% CI 0.01 to 0.18) higher and LDL cholesterol 0.10 mmol/L (95% CI 0.02 to 0.18) higher than in children who never or hardly ever consumed takeaway meals. There were no differences in insulin resistance, blood pressure or any of the other risk markers by frequency of takeaway meal consumption. The differences in total cholesterol and LDL cholesterol were similar in different ethnic groups (tests for interaction all P >0.05) and were little affected by additional adjustment for fat mass index, remaining statistically significant (data not presented).

**Table 1:** CHD and type 2 diabetes risk markers by frequency of takeaway meal consumption (1948 children)

	Frequency of takeaway meal consumption			Differences/ % differences*≥1week − never or hardly ever	P value (trend)
	Never, hardly ever (n=499)	<1 per week (n=894)	≥1 per week (n=555)		
	Mean (95% CI)	Mean (95% CI)	Mean (95% CI)		
Fat mass index (kg/m^5^)*	2.01 (1.95 to 2.08)	2.10 (2.05 to 2.15)	2.11 (2.05 to 2.18)	5.06 (0.53 to 9.79)	0.03
Sum of skinfolds (mm)*	39.8 (38.1 to 41.6)	41.8 (40.4 to 43.2)	42.3 (40.5 to 44.1)	6.14 (0.05 to 12.60)	0.05
Total cholesterol (mmol/L)	4.44 (4.37 to 4.50)	4.51 (4.46 to 4.56)	4.53 (4.47 to 4.59)	0.09 (0.00 to 0.18)	0.04
LDL cholesterol (mmol/L)	2.58 (2.52 to 2.64)	2.66 (2.61 to 2.70)	2.68 (2.62 to 2.73)	0.10 (0.02 to 0.18)	0.01
HDL cholesterol (mmol/L)	1.53 (1.50 to 1.56)	1.53 (1.51 to 1.56)	1.54 (1.51 to 1.57)	0.01 (−0.02 to 0.05)	0.47
Systolic BP (mm Hg)	104.5 (103.4 to 105.6)	104.6 (103.7 to 105.5)	103.4 (102.3 to 104.5)	−1.12 (−2.41 to 0.17)	0.08
Diastolic BP (mm Hg)	62.5 (61.6 to 63.4)	62.8 (62.1 to 63.6)	62.2 (61.3 to 63.1)	−0.27 (−1.40 to 0.87)	0.63
Insulin (mmol/L)*	7.17 (6.72 to 7.66)	7.28 (6.90 to 7.69)	7.31 (6.86 to 7.78)	1.84 (−5.48 to 9.72)	0.64
HbA1c (%)*	5.27 (5.24 to 5.30)	5.25 (5.23 to 5.27)	5.28 (5.25 to 5.31)	0.28 (−0.49 to 1.06)	0.45
Glucose (mmol/L)*	4.46 (4.43 to 4.49)	4.44 (4.42 to 4.47)	4.48 (4.45 to 4.51)	0.62 (−0.25 to 1.50)	0.15
Triglycerides (mmol/L)*	0.83 (0.80 to 0.86)	0.83 (0.81 to 0.85)	0.81 (0.78 to 0.84)	−2.60 (−6.99 to 2.00)	0.26

Adjusted means and 95% CI are adjusted for age (quartiles), sex, month, ethnic group, socioeconomic status and school (random effect). Missing values for: systolic and diastolic BP,^3^ insulin,^8^ HbA1c and^19^ glucose.^14^

*Geometric means are presented for variables that were log transformed prior to undertaking the analysis.

BP, blood pressure; CHD, coronary heart disease; HbA1c, haemoglobin A1c; HDL, high-density lipoprotein; LDL, low-density lipoprotein.

Associations between frequency of takeaway meal consumption and dietary nutrient intakes, estimated using the 24-hour recall data, were investigated (table 2). Higher reported takeaway meal frequency was associated with higher mean intakes for total energy, energy density, total fat, saturated fat and monounsaturated fat (as percentages of energy) increased (all P<0.0001), while intakes of starch and protein (also expressed as percentages of energy) were lower. Higher reported takeaway meal frequency was also associated with lower intakes of vitamin C, iron, calcium and folate. These associations were not materially affected by the exclusion of 180 children who reported having a takeaway evening meal in their 24-hour recall (data not presented). Finally, the nutrient contents of the evening meal included in the 24-hour dietary recall were compared, according to whether meals were prepared at home or obtained from a takeaway restaurant (table 3). The evening meals obtained from takeaway restaurants were more energy dense, with higher energy content and higher total and saturated fat content than meals prepared at home.

**Table 2:** The nutrient composition of diets by frequency of takeaway meal consumption in 1948 children

	Frequency of takeaway meal consumption			Differences/ % differences* ≥1week − never or hardly ever	P value (trend)
	Never, hardly ever (n=499)	<1 per week (n=894)	≥1 per week (n=555)		
	Mean (95% CI)	Mean (95% CI)	Mean (95% CI)		
Total energy (kcals)	1723 (1678 to 1767)	1850 (1816 to 1885)	1950 (1908 to 1993)	228 (169 to 286)	<0.0001
Energy density	6.7 (6.6 to 6.9)	7.0 (6.9 to 7.1)	7.3 (7.1 to 7.4)	0.5 (0.3 to 0.7)	<0.0001
Fat % energy	33.5 (32.9 to 34.0)	34.4 (34.0 to 34.8)	34.8 (34.3 to 35.4)	1.4 (0.6 to 2.2)	<0.001
Saturated fat % energy	12.2 (11.9 to 12.5)	12.7 (12.4 to 12.9)	13.1 (12.8 to 13.4)	0.9 (0.5 to 1.3)	<0.0001
Monounsaturated fat % energy	11.0 (10.7 to 11.3)	11.5 (11.2 to 11.7)	11.7 (11.4 to 11.9)	0.6 (0.3 to 1.0)	<0.001
Polyunsaturated fat % energy	6.6 (6.3 to 6.9)	6.6 (6.4 to 6.8)	6.4 (6.1 to 6.7)	−0.2 (−0.6 to 0.2)	0.27
Carbohydrates % energy	52.6 (52.0 to 53.2)	52.0 (51.5 to 52.4)	51.8 (51.2 to 52.4)	−0.8 (−1.6 to 0.0)	0.06
Sugars % energy	22.3 (21.6 to 22.9)	22.6 (22.1 to 23.1)	22.6 (21.9 to 23.2)	0.3 (−0.5 to 1.1)	0.48
Starch % energy	29.8 (29.2 to 30.4)	28.9 (28.4 to 29.4)	28.7 (28.1 to 29.3)	−1.1 (−1.8, to 0.4)	0.004
Non-starch polysaccharides (g)	11.6 (11.1 to 12.0)	11.9 (11.5 to 12.3)	12.0 (11.5 to 12.4)	0.4 (−0.2 to 0.9)	0.20
Protein % energy	13.6 (13.3 to 13.9)	13.3 (13.1 to 13.6)	13.0 (12.7 to 13.3)	−0.6 (−1.0, to 0.2)	0.003
Vitamin B_12_ (µg)*	3.0 (2.8 to 3.3)	2.9 (2.8 to 3.1)	2.9 (2.7 to 3.1)	8.2 (−1.3 to 18.7)	0.22
Vitamin C (mg)*	83.7 (77.8 to 90.1)	82.6 (77.9 to 87.5)	76.2 (71.0 to 81.8)	−0.6 (−9.6 to 9.4)	0.05
Iron (mg)*	8.9 (8.7 to 9.2)	8.9 (8.7 to 9.1)	8.6 (8.4 to 8.9)	7.3 (2.7 to 12.0)	0.09
Calcium (mg)*	748 (722 to 776)	719 (698 to 741)	681 (657 to 705)	2.4 (−2.9 to 8.1)	<0.0001
Folate (µg)*	207 (199 to 215)	196 (191 to 203)	195 (188 to 203)	3.2 (−1.9 to 8.6)	0.02

Means and 95% CIs are adjusted for age (quartiles), sex, month, ethnic group, socioeconomic status and school (random effect), micronutrients are also adjusted for total energy intake.

*Geometric means are presented for variables that were log transformed prior to undertaking the analysis.

**Table 3:** Nutrient composition of the 24-hour recall-based evening meal by source in 1793 children who provided information on evening meal source

Nutrient composition of evening meal	Source of evening meal		P value (no difference)
	Home (n=1637)	Takeaway restaurant (n=156)	
	Mean (95% CI)	Mean (95% CI)	
Total energy (kcals)	527 (510 to 543)	686 (643 to 728)	<0.0001
Energy density	4.3 (4.2 to 4.4)	5.8 (5.4 to 6.2)	<0.0001
Fat % energy	33.3 (32.7 to 34.0)	39.7 (37.6 to 41.8)	<0.0001
Saturated fat % energy	11.3 (10.9 to 11.6)	15.6 (14.5 to 16.6)	<0.0001
Monounsaturated fat % energy	11.4 (11.1 to 11.7)	13.1 (12.2 to 14.0)	0.0003
Polyunsaturated fat % energy	6.8 (6.5 to 7.0)	5.6 (4.8 to 6.4)	0.005
Carbohydrates % energy	50.6 (49.8 to 51.3)	46.1 (43.8 to 48.5)	0.0004
Sugars % energy	30.5 (29.7 to 31.4)	27.4 (25.2 to 29.7)	0.01
Starch % energy	19.6 (18.6 to 20.6)	18.0 (15.4 to 20.7)	0.25
Non-starch polysaccharides (g)	3.7 (3.5 to 3.9)	3.8 (3.3 to 4.3)	0.74
Protein % energy	16.1 (15.6 to 16.5)	13.8 (12.6 to 15.0)	0.0003
Vitamin B_12_ (µg)*	1.0 (1.0 to 1.1)	1.2 (1.0 to 1.4)	0.15
Vitamin C (mg)*	16.6 (15.6 to 17.7)	13.7 (11.2 to 16.8)	0.08
Iron (mg)*	2.1 (2.0 to 2.1)	2.3 (2.0 to 2.6)	0.10
Folate (µg)*	40.2 (38.6 to 41.8)	47.7 (42.0 to 54.2)	0.01
Calcium (mg)*	123.9 (117.5 to 130.5)	124.4 (107.6 to 143.7)	0.96

Adjusted means and 95% CIs are adjusted for age (quartiles), sex, month, ethnic group, socioeconomic status and school (random effect).

*Geometric means are presented for variables that were log transformed prior to undertaking the analysis.

In further analyses examining whether the higher total cholesterol and LDL cholesterol levels observed in the children who consumed a takeaway meal ≥1 a week could be accounted for by their higher intake of saturated fat % energy, and taking account of imprecision in saturated fat intakes using classical error models, the differences in total and LDL cholesterol were reduced by ~20% and ~13%, respectively; the difference in total cholesterol became statistically non-significant. The differences in total cholesterol and LDL cholesterol were also slightly reduced once total energy intakes were included in the models. Sensitivity analysis found similar patterns of association in boys and girls and no further changes after additional adjustment for pubertal status.

## Discussion

In this study of UK primary school children of diverse ethnic origins, children who regularly consumed takeaway meals had higher circulating total cholesterol and LDL cholesterol concentrations and higher levels of adiposity than children who did not; they also had more energy-dense diets with higher intakes of total energy, fat and saturated fat and lower micronutrient intakes (including vitamin C and folate). In an analysis comparing nutrient intakes in the previous evening meal in relation to its source, meals from a takeaway restaurant were more energy dense and had much higher fat and saturated fat contents than meals prepared at home.

These results are consistent with previous studies which have shown that children who regularly eat takeaway food have higher energy, fat and saturated fat intakes,^25 26^ and consume less fruit and vegetables.^9 10 27^ The significantly lower starch, protein and calcium intakes in this group may also be adversely affecting the health of these children, particularly if these dietary patterns are sustained. To our knowledge, no previous study has reported on the associations between frequency of takeaway meal consumption and risk markers for CHD and type 2 diabetes in children. Our findings are consistent with previous reports in young adults and adolescents in the USA and UK showing that frequent fast food consumption was associated with higher levels of body fatness.^7 28^ However, in contrast with previous reports in young American and Australian adults, we did not find evidence that frequent fast food consumption was associated with evidence of insulin resistance and hyperglycaemia.^4 29^ We did however observe associations between frequent takeaway meal consumption and higher total and LDL cholesterol, which were consistent with our own observations and a previous report^25^ showing associations between fast food consumption and high saturated fat intake. Moreover, in the light of the well-documented association between LDL cholesterol and CHD risk,^30^ our findings are also consistent with evidence that greater consumption of takeaway meals is associated with higher CHD risk.^6^

The analyses presented in this paper related frequency of self-reported takeaway meal consumption to a wide range of CHD and type 2 diabetes risk factors, in a large ethnically diverse population. By including a measure of socioeconomic status based on parental occupation in our models, we were able to take an important potential confounding factor into account. Although we were not able to examine the validity of self-reported takeaway meal consumption directly, this is supported by the expected gender and socioeconomic patterns^27 31^ and the expected absence of appreciable ethnic differences.^12^ The validity of the dietary data, based on the multiple pass 24-hour recall method, has been supported by previous analyses.^32^ The cross-sectional nature of the current study makes it a particularly appropriate design for investigating short-term associations between diet and blood-based risk markers,^33^ although it remains difficult to establish the direction of causality and to interpret differences in overall nutrient intakes between the different takeaway meal frequency groups and the underlying mechanisms. Further research is needed to establish the extent of causality between high consumption of takeaway meals and CHD risk markers in children.

The higher total cholesterol and LDL cholesterol concentrations observed in the frequent takeaway meal group, if sustained, are sufficiently large to increase long-term CHD risk by ~10%.^30^ However, the estimates of the excess saturated fat intake associated with consumption of one takeaway meal per week (table 3) would be insufficient to account for more than about one-third of the observed overall difference in saturated fat intake (table 2) and more than about one-seventh of the difference in LDL cholesterol between the highest and lowest takeaway intake groups (table 1). Although this could reflect systematic under-reporting of takeaway frequency in the highest frequency group, a more likely explanation is that these groups have markedly different dietary nutrient intakes in general, with particularly adverse dietary patterns (including high energy and high saturated fat intake) in the highest takeaway meal frequency group. The direction of causality in this association remains unclear, however there is evidence that the regular consumption of takeaway foods affects taste preferences, particularly for high fat, energy-dense foods which influence overall dietary nutrient patterns.^34 35^ These findings highlight the need for further investigation (particularly prospective studies) of the associations between takeaway meals, dietary patterns and risk markers for chronic disease both in children and also in adolescents, who have much higher takeaway consumption rates.^12^ This issue is particularly important in the context of the rising prevalence of takeaway meal outlets in many neighbourhoods (increasing more rapidly in more deprived areas^36^), which is itself an important determinant of increased takeaway meal consumption.^1^ Technological advances have also meant that it is now much easier to order and have delivered take away foods, resulting in an increase in consumption and although healthier takeaway foods are becoming available, this may well be strongly socially patterned.

These results suggest that further increases in takeaway meal consumption (and marketing directed at encouraging such increases) are likely to have adverse public health consequences and should be actively discouraged. The government should be considering health protection initiatives to reverse the current trends in takeaway meal consumption, in the context of broader efforts to improve childhood diet and nutrition in home and school settings.
