# Supplementary material for: Takeaway meal consumption and risk markers for coronary heart disease, type 2 diabetes and obesity in children aged 9–10 years: a cross-sectional study
Source: Arch Dis Child. 2017 Dec 3;103(5):431–6. doi: 10.1136/archdischild-2017-312981 (PMC5916105; doi:10.1136/archdischild-2017-312981)
Supplement: Supplementary Table 1 [file archdischild-2017-312981supp001.pdf]

Supplementary Table 1: Frequency of takeaway meal consumption in 1948 children; by sex, ethnic group and socioeconomic status

	Frequency of takeaway meal consumption						
	Never, hardly ever		< 1 per week		≥1 per week		
	Frequency	(%)	Frequency	(%)	Frequency	(%)	p (no difference)
All children (n=1948)	499	(26)	894	(46)	555	(28)	
Boys	217	(24)	411	(44)	297	(32)	
Girls	282	(28)	483	(47)	258	(25)	0.001
white European	135	(28)	226	(48)	114	(24)	
Black African Caribbean	106	(21)	232	(47)	158	(32)	
South Asian	121	(24)	223	(45)	151	(31)	
Other ethnicity	137	(29)	213	(44)	132	(27)	0.72
Managerial	135	(26)	258	(51)	115	(23)	
Intermediate	118	(25)	232	(49)	127	(26)	
Routine & manual	141	(25)	247	(43)	181	(32)	
Inactive	85	(28)	124	(40)	99	(32)	
Unclassified/missing	20	(23)	33	(38.5)	33	(38.5)	0.008
